# Alarmins HMGB1, IL-33, S100A7, and S100A12 in Psoriasis Vulgaris

**DOI:** 10.1155/2020/8465083

**Published:** 2020-04-15

**Authors:** Pavel Borsky, Zdenek Fiala, Ctirad Andrys, Martin Beranek, Kvetoslava Hamakova, Andrea Malkova, Tereza Svadlakova, Jan Krejsek, Vladimir Palicka, Lenka Borska, Vit Rehacek

**Affiliations:** ^1^Institute of Hygiene and Preventive Medicine, Faculty of Medicine in Hradec Kralove, Charles University, Hradec Kralove 500 03, Czech Republic; ^2^Institute of Pathological Physiology, Faculty of Medicine in Hradec Kralove, Charles University, Hradec Kralove 500 03, Czech Republic; ^3^Institute of Clinical Immunology and Allergology, University Hospital and Faculty of Medicine in Hradec Kralove, Charles University, Hradec Kralove 500 03, Czech Republic; ^4^Institute of Clinical Biochemistry and Diagnostics, University Hospital and Faculty of Medicine in Hradec Kralove, Charles University, Hradec Kralove 500 03, Czech Republic; ^5^Clinic of Dermal and Venereal Diseases, University Hospital Hradec Kralove, Hradec Kralove 500 03, Czech Republic; ^6^Transfusion Center, University Hospital Hradec Kralove, Hradec Kralove 500 03, Czech Republic

## Abstract

**Background:**

Psoriasis vulgaris is a chronic autoimmune disease associated with systemic inflammation. Increased levels of numerous cytokines, chemokines, growth factors, and other molecules were found in the skin and in the circulation of psoriatic patients. Alarmins, also known as danger signals, are intracellular proteins, which are released to an extracellular space after infection or damage. They are the markers of cell destructive processes.

**Objective:**

The aim of the present study was to evaluate the suitability of selected alarmins (HMGB1, IL-33, S100A7, and S100A12) as potential biomarkers of severity of psoriasis and to explore possible relationships between these proteins for the purpose of better understanding their roles in the immunopathology of psoriasis.

**Methods:**

The serum levels of selected alarmins were measured in 63 psoriatic patients and 95 control individuals. The levels were assessed by the ELISA technique using commercial kits. The data were statistically processed with MedCalc version 19.0.5.

**Results:**

In psoriatic patients, we found significantly increased levels of HMGB1 (*p* < 0.05), IL-33 (*p* < 0.01), S100A7 (*p* < 0.0001), and S100A12 (*p* < 0.0001). In addition, we found a significant relationship between HMGB1 and S100A7 (Spearman′s rho = 0.276, *p* < 0.05) in the patients and significant relationship between HMGB1 and IL-33 in the controls (Spearman′s rho = 0.416, *p* < 0.05). We did not find any relationship between observed alarmins and the disease severity.

**Conclusions:**

The alarmins HMGB1, IL-33, S100A7, and S100A12 were significantly elevated in the serum of patients, which states the hypothesis that they play specific roles in the immunopathology of psoriasis. However, we have not yet found a relationship between observed alarmins and the disease severity. The discovery of the relationship between HMGB1 and S100A7 is a novelty that should be studied in the future to further clarify its role and importance.

## 1. Introduction

Psoriasis vulgaris is a chronic autoimmune multifactorial disease associated with systemic inflammation. Its pathogenesis constitutes of enhanced proliferation and a shortened duration of maturation of keratinocytes; perivascular infiltration of T cells, dendritic cells, macrophages, and neutrophilic granulocytes; and imbalance in apoptotic pathways [1–3].

There are increased levels of numerous cytokines, chemokines, growth factors, and other molecules in the skin and in the circulation of patients with psoriasis [4]. Alarmins are intracellular proteins, which are released to an extracellular space after infection or damage. They are considered biomarkers of cell destructive processes. Extracellular alarmins confer inflammatory signaling pathways via Pattern Recognition Receptors (PRRs) that are engaged in host defense which results in initiation of innate and adaptive immune responses, triggering inflammation or tissue repair [5, 6]. They can enhance the adaptive immune response through their effects on antigen-presenting cells, including dendritic cells. The perseverant release of alarmins may lead to proliferation of antigen-specific T lymphocytes and promote the polarization toward a Th1 phenotype. This effect initiates the local hyperinflammatory environment, which is present in psoriasis [1, 7].

Elevated levels of alarmins can be seen in acute and chronic inflammatory conditions as well as certain types of cancer, especially that High Mobility Group Box 1 (HMGB1) and IL-33 are known to play roles in antitumor immune response [8, 9].

In correspondence to inflammatory biomarkers such as C-reactive protein (CRP) or erythrocyte sedimentation rate (ESR), the levels of alarmins correlate with disease activity in several inflammatory conditions, such as sepsis, rheumatoid arthritis, Kawasaki disease, or idiopathic bowel disease. Alarmins were also found elevated after trauma, surgery, or acute coronary syndrome. They show important advantages over traditional clinical and laboratory markers for specific indications, probably due to their local expression and release in direct response to tissue damage. Their serum levels were found to better correlate with the disease activity, especially in the diseases with joint disabilities (rheumatoid arthritis) [10, 11]. HMGB1, IL-33, S100A7, and S100A12 were chosen as the most promising alarmins according to the literature, because they were reported to be elevated in various autoimmune diseases, yet their role in pathophysiology of psoriasis is still unclear [8, 12, 13].

HMGB1, also known as amphoterin, is an evolutionary ancient and highly conservative damage-associated molecular pattern molecule. While in nucleus, HMGB1 binds and bends the DNA helix to help to regulate nuclear biochemical transactions. After a damage of a cell, HMGB1 can get to the extracellular matrix, where it functions as a common signal of tissue injury [14, 15]. Recent studies have found that HMGB1 plays significant roles in many parts of human metabolome. There has been an ongoing research finding connections between HMGB1 and immunopathology of disorders and traumas in various body systems. Its role in ischemic stroke was described by Ye et al. [16]. Its complex function in cardiomyocyte senescence and cardiac inflammatory injury was described by Lu et al. [17]. However, there was not an excessive research done in the pathogenesis of psoriasis and HMBG1.

IL-33 is an inflammatory member of IL-1 superfamily of cytokines released from cells undergoing necrosis [18]. IL-33 has been described as an epithelial “alarmin” defense system. It is also released after cellular damage through the activation of various immune cells (with ST2 receptor), which leads to the production of various molecules, including IL-33. The IL-33-induced production of proinflammatory cytokines plays an important role as a bridge between innate and adaptive immune responses in allergic diseases [19, 20]. Its higher serum levels were found in various immunopathological conditions, such as rheumatoid arthritis, inflammatory bowel disease, or atopic dermatitis [20].

S100A7, a small calcium-binding protein from the S100 group of alarmins, is an antimicrobial peptide and signaling molecule which regulates cellular functions [21]. Its role in epidermal differentiation remains largely unknown. However, the evidence of S100A7 involvement in the pathogenesis of psoriasis is growing. It was found highly expressed in keratinocytes of psoriatic lesions and named psoriasin [22–24].

S100A12 is also a member of the S100 family of proteins, who as well as the others shares EF-hand domains involved in binding calcium. It can even be actively secreted by activated granulocytes in order to potent chemotactic activity comparable with other strongly potent chemotactic agents [25]. According to the study of Wilsmann-Theis et al., S100A12 is the most promising marker of psoriasis disease activity [26].

The aims of presented study were to evaluate the suitability of the levels of HMGB1, IL-33, S100A7, and S100A12 as potential biomarkers of severity of psoriasis and to explore possible relationships between these proteins for the purpose of understanding of their roles in the immunopathology of psoriasis.

## 2. Materials and Methods

### 2.1. Observed Groups of Persons

The study group consisted of 63 patients (30 females and 33 males) with acute skin manifestation of psoriasis vulgaris. Median age was 43 years, range 15-80 years. The intensity/severity of the psoriasis was calculated from basic characteristics of the disease status (desquamation, erythema, and skin infiltration) and expressed as the PASI score (Psoriasis Area and Severity Index) [27]. The patients were without any immunomodulation therapy for at least two weeks before the enrollment to the study. None of them had systemic psoriasis therapy ever applied. Their exposure history was examined using a questionnaire. The patients with acute infections, psoriatic arthritis, or other chronic inflammatory diseases were excluded.

Control group data were collected from a group of 95 healthy blood donors (45 females and 50 males), whose median age was 47 years, range 21-65 years. Neither the patients nor the controls had been treated by any drugs influencing inflammatory response.

All the samples were collected throughout the time period of year (2018).

### 2.2. Blood Collection

The samples of peripheral blood from the patients with psoriasis and from the control group were collected from the cubital vein using Vacutainer sampling tubes (Becton Dickinson) and incubated for 30 minutes at room temperature. Blood serum was isolated by centrifugation for 10 minutes at 1300 g (2500 rpm) and stored under –70°C until analysis. Repeated thawing and freezing were avoided.

### 2.3. Laboratory Analysis in Serum

Serum levels of S100A7 and S100A12 were assessed with enzyme-linked immunosorbent assays (ELISAs) by means of the Enzyme-linked Immunosorbent Assay Kit For S100 Calcium Binding Protein A7 (S100A7) and with the Enzyme-linked Immunosorbent Assay Kit For S100 Calcium Binding Protein A12 (S100A12) according to the manufacturer's instructions, respectively. Both kits were made by Cloud-Clone Corp., Houston, TX, USA). The sensitivities of the kits were 0.050 ng/ml (S100A7) and 0.031 ng/ml (S100A12). Serum samples were 10-fold (S100A7) and 50-fold (S100A12) diluted.

Serum concentrations of human HMGB1 were determined by sandwich enzyme-linked immunosorbent assay technique (ELISA) with Human HMGB1 ELISA kit (IBL International GmbH, Hamburg, Germany) according to the manufacturer's instructions. The limit of detection of HMGB1 was 0.20 ng/ml.

Concentrations of IL-33 were determined in serum samples using a commercial ELISA Quantikine ELISA Human IL-33 Immunoassay (R&D System, Inc., Minneapolis, MN). The limit of detection of the kit was 0.357 pg/ml.

Absorbance values were read at 450 nm/620 nm by the Multiskan RC ELISA reader (Thermo Fisher Scientific, USA).

### 2.4. Statistical Analysis

All data were statistically processed with MedCalc version 19.0.5. Based on the D'Agostino-Pearson test for the data distribution, either the parametric or nonparametric test was used to ensure the proper test sensitivity. Associations between parameters were evaluated by Pearson's correlation test and Spearman's rank correlation test. Intergroup differences were assessed using Student's *t*-test or the Mann–Whitney test. The differences were considered statistically significant when the probability level (*p*) was below the alpha level of 0.05.

### 2.5. Approval of the Ethics Committee

The study was conducted in accordance with the Declaration of Helsinki, and the study protocol was approved by the Ethics Committee of the University Hospital in Hradec Kralove, the Czech Republic (project identification code: PROGRES Q40-09, Q40-10, and Q40-11; reference number: 201705 183P; date of approval: May 2, 2017). Informed written consent was obtained from all persons.

## 3. Results

The median of PASI score in the group of patients was 17.4 (*N* = 63; interquartile range 13.3-21.0).

**Figure 1:**
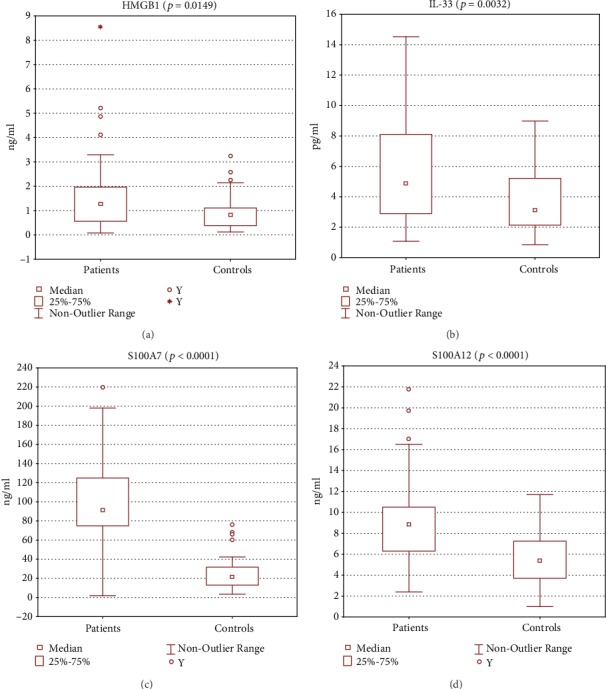
All the measured parameters were elevated in the group of patients when compared to healthy controls. (a) High Mobility Group Box 1 (HMGB1; patients, *N* = 51, median 1.250; controls, *N* = 35, median 0.810; *p* < 0.05, Mann–Whitney test). (b) IL-33 (patients, *N* = 51, median 4.890; controls, *N* = 35, median 3.110; *p* < 0.01, Student's *t*-test). (c) S100A7 (patients, *N* = 63, median 91.0; controls, *N* = 60; median 21.7; *p* < 0.0001, Mann–Whitney test). (d) S100A12 (patients, *N* = 63, median 8.80; controls, *N* = 60; median: 5.40; *p* < 0.0001, Mann–Whitney test).

The serum levels of all observed alarmins were significantly elevated in the group of patients. The level of HMGB1 (ng/ml; patients, *N* = 51, median 1.250, interquartile range 0.608-1.942; controls, *N* = 35, median 0.810, interquartile range 0.390-1.092; *p* < 0.05; Figure 1(a)). The level of IL-33 (pg/ml; patients, *N* = 51, median 4.890, interquartile range 2.945-7.962; controls, *N* = 35, median 3.110, interquartile range 2.158-5.200; *p* < 0.01; Figure 1(b)). The level of S100A7 (ng/ml; patients, *N* = 63, median 91.0, interquartile range 75.0-124.0; controls, *N* = 60; median 21.7, interquartile range 13.0-31.7; *p* < 0.0001; Figure 1(c)). The level of S100A12 (ng/ml; patients, *N* = 63, median 8.80, interquartile range 6.35-10.50; controls, *N* = 60; median: 5.40; interquartile range 3.70-7.25; *p* < 0.0001; Figure 1(d)).

**Figure 2:**
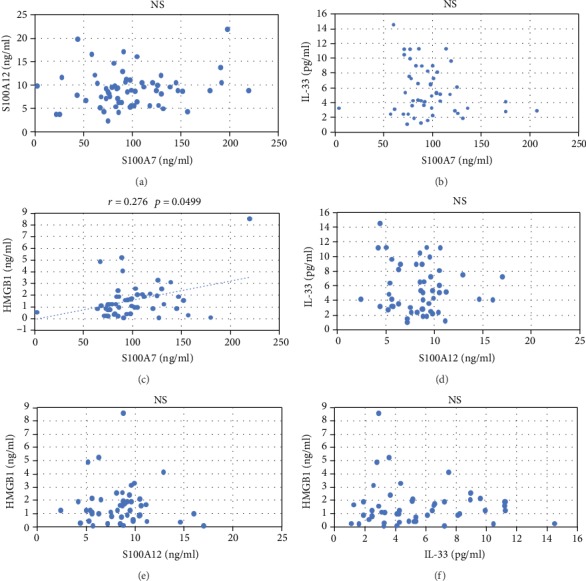
This figure depicts possible correlations between each of the alarmins in the group of patients. Statistical significance was found using Spearman's rank correlation test between HMGB1 and S100A7 (*r* = 0.276, *p* = 0.0499).

We found a significant relationship between HMGB1 and S100A7 (Spearman′s rho = 0.276, *p* < 0.05) in the group of patients (Figure 2(c)) and a significant relationship between HMGB1 and IL-33 in the group of controls (Spearman′s rho = 0.416, *p* < 0.05). No statistically significant correlation was found between PASI and any of the alarmins.

## 4. Discussion

Our results showed that psoriasis is a disease in which the levels of all investigated alarmins are affected. However, the magnitude of the disease was not found crucial for the change of these parameters. The role of alarmins remains unclear and should be thoroughly researched in the future. It is yet unknown whether the alarmins are a side product of the autoimmune processes or whether they are significantly contributing to the cascade of deeper exacerbation of the psoriasis.

The present study found higher levels of HMGB1 in the patients than in the controls (*p* < 0.05). In concordance with our results, Strohbuecker et al. and Bergmann et al. previously showed that HMGB1 is significantly increased in the serum of patients with psoriasis; they have also suggested that the presence of HMGB1 may impact the composition of chronic inflammation in psoriasis which might have implications for Treg and Th17cells [15, 28]. Unlike their study, we have not found any relationship between the severity of disease (PASI) and HMGB1. Higher serum levels of HMGB1 were also found in several other autoimmune diseases, such as neuromyelitis optica, lupus nephritis, or rheumatoid arthritis [29–31]. These findings suggest that HMGB1 might play a role in the immunopathology of psoriasis as well as other autoimmune diseases.

We have confirmed that patients with psoriasis vulgaris have higher levels of alarmin IL-33 than healthy controls (*p* < 0.01). This finding is consistent with the study from Mitsui et al., who measured the levels of serum IL-33 in patients with psoriasis vulgaris, psoriatic arthritis, or pustular psoriasis and found them to be higher than in controls. They have also found that the levels of IL-33 correlated to the TNF-alpha levels. No correlation between IL-33 and PASI score was found neither in our nor their study [32]. Higher levels of IL-33 in patients and no correlation with PASI were also found in study from Li et al. [33]. However, a recent study from Sehat et al. found IL-33 serum levels to be equal to those in controls and they found a correlation between PASI and IL-33 [18]. These contradictory findings suggest that more research deeply focused on the immunopathology of IL-33 in psoriasis is needed. Higher serum levels of IL-33 were also found in atopic dermatitis, generalized vitiligo, or Still's disease [20, 34, 35].

In our study, we have found higher levels of S100A7 (psoriasin) in the patients than in healthy controls which is in concordance with the results of study of Gambichler et al. Nevertheless, we have not found any significant relationship of S100A7 with PASI or did Gambichler et al. [36]. However, the team of Anderson et al. has found rather lower systemic levels of psoriasin in patients. The reason for this was explained by the presence of antibodies against psoriasin, and neither psoriasin nor the antibodies were suggested valid biomarkers of psoriasis [21]. The mechanism of its action in psoriasis pathogenesis is to prime keratinocytes and neutrophils for enhanced production of proinflammatory cytokines (TNF-*α*, IL-6, and IL-8). It also appears to induce angiogenesis [37–39].

Another study published in 2013 found that psoriasin correlated to the PASI of patients and also to their obesity [40]. The significance of psoriasin was emphasized by Awad et al. demonstrating an association of psoriasin levels with intima-media thickness of patients and indicating a potential link between psoriasis and atherosclerosis [41]. In addition, higher serum levels of psoriasin were also found in patients with systemic sclerosis [42].

A previous study of S100A7, S100A8, S100A9, and S100A12 showed that all these alarmins were significantly higher in psoriatic patients than in controls. It was also suggested that S100A12, also called calgranulin-c, might be the most promising bioindicator of psoriasis with the closest association with the disease activity evaluated by PASI [26]. Our data deeper confirm this statement; however, we have not found any significant relationship between calgranulin-c and the disease severity, which supports the idea of conducting more research into S100A12 before a conclusion on the quality of psoriasis biomarker can be made.

In the group of patients, we have found a significant relationship between HMGB1 and psoriasin (Spearman′s rho = 0.276, *p* < 0.05). This relationship has never been published before in any disease, and it will need more research to clarify its potential role in the immunopathology of psoriasis. A feasible reason of this correlation could be that these two alarmins are released at the same time during the psoriatic autoimmune process or that they are released after specific cell death. A possible cause-effect relationship should not be excluded as a hypothesis. In the group of healthy controls, we observed a significant relationship between HMGB1 and IL-33 (Spearman′s rho = 0.416, *p* < 0.05). This correlation could be attributed to the suspected relationship between upregulation of IL-33 and the release of HMGB1.

No significant correlation was found between the selected alarmins and PASI. The PASI score is an objective indicator of disease severity, which was found to correlate with markers of inflammation, such as CRP or TNF-*α* [43]. However, we did not find a correlation of these markers with PASI in our previous studies [3, 44]. This might be attributed to the fact that the markers of inflammation are influenced by many variables.

A limitation of the study is the fact that the samples were obtained from patients with acute exacerbated manifestation of psoriasis. The disease is chronic, and we should not apply this knowledge of elevated alarmins to the periods of remission without more research. The study suffers from selection bias. A relatively low number of patients in the study are determined by the strict exclusion criteria and low number of patients requiring doctor visits in the population. A limiting factor of result interpretation is the wide interval of patients' age.

## 5. Conclusions

In summary, the study found all four monitored immunoparameters significantly increased in the serum of psoriatic patients. It is obvious that psoriasis affects their levels. However, we have not yet found a relationship between observed alarmins and the disease severity. This suggests that more research should be done to better understand whether alarmins are a side product of the damage or whether they play a significant role in the autoimmune cascade of psoriasis exacerbation. The discovery of the relationship between HMGB1 and S100A7 is a novelty that should be studied in the future to further clarify its role and importance.

## Data Availability

The data used to support the findings of this study are available from the corresponding author upon request.
